# Health Care Professionals’ Beliefs About Using Wiki-Based Reminders to Promote Best Practices in Trauma Care

**DOI:** 10.2196/jmir.1983

**Published:** 2012-04-19

**Authors:** Patrick Michel Archambault, Andrea Bilodeau, Marie-Pierre Gagnon, Karine Aubin, André Lavoie, Jean Lapointe, Julien Poitras, Sylvain Croteau, Martin Pham-Dinh, France Légaré

**Affiliations:** ^1^Centre de santé et de services sociaux Alphonse-Desjardins (Centre hospitalier affilié universitaire de Lévis)Lévis, QCCanada; ^2^Département de médecine familiale et de médecine d’urgenceFaculté de médecineUniversité LavalQuebec, QCCanada; ^3^Traumatologie – Urgence – Soins intensifsCentre de recherche FRQS du CHA universitaire de QuébecQuebec, QCCanada; ^4^Centre de recherche du Centre hospitalier universitaire de Québec (CRCHUQ)Quebec, QCCanada; ^5^Faculté des sciences infirmièresUniversité LavalQuebec, QCCanada; ^6^Institut national d’excellence en santé et services sociauxMontreal, QCCanada; ^7^Hôpital de GatineauGatineau, QCCanada

**Keywords:** Wiki, Collaborative writing applications, Web 2.0, traumatic brain injury, interprofessional collaboration, reminders, computerized clinical decision-support system, knowledge translation, evidence-based medicine, theory of planned behavior

## Abstract

**Background:**

Wikis are knowledge translation tools that could help health professionals implement best practices in acute care. Little is known about the factors influencing professionals’ use of wikis.

**Objectives:**

To identify and compare the beliefs of emergency physicians (EPs) and allied health professionals (AHPs) about using a wiki-based reminder that promotes evidence-based care for traumatic brain injuries.

**Methods:**

Drawing on the theory of planned behavior, we conducted semistructured interviews to elicit EPs’ and AHPs’ beliefs about using a wiki-based reminder. Previous studies suggested a sample of 25 EPs and 25 AHPs. We purposefully selected participants from three trauma centers in Quebec, Canada, to obtain a representative sample. Using univariate analyses, we assessed whether our participants’ gender, age, and level of experience were similar to those of all eligible individuals. Participants viewed a video showing a clinician using a wiki-based reminder, and we interviewed participants about their behavioral, control, and normative beliefs—that is, what they saw as advantages, disadvantages, barriers, and facilitators to their use of a reminder, and how they felt important referents would perceive their use of a reminder. Two reviewers independently analyzed the content of the interview transcripts. We considered the 75% most frequently mentioned beliefs as salient. We retained some less frequently mentioned beliefs as well.

**Results:**

Of 66 eligible EPs and 444 eligible AHPs, we invited 55 EPs and 39 AHPs to participate, and 25 EPs and 25 AHPs (15 nurses, 7 respiratory therapists, and 3 pharmacists) accepted. Participating AHPs had more experience than eligible AHPs (mean 14 vs 11 years; *P *= .04). We noted no other significant differences. Among EPs, the most frequently reported advantage of using a wiki-based reminder was that it refreshes the memory (n = 14); among AHPs, it was that it provides rapid access to protocols (n = 16). Only 2 EPs mentioned a disadvantage (the wiki added stress). The most frequently reported favorable referent was nurses for EPs (n = 16) and EPs for AHPs (n = 19). The most frequently reported unfavorable referents were people resistant to standardized care for EPs (n = 8) and people less comfortable with computers for AHPs (n = 11). The most frequent facilitator for EPs was ease of use (n = 19); for AHPs, it was having a bedside computer (n = 20). EPs’ most frequently reported barrier was irregularly updated wiki-based reminders (n = 18); AHPs’ was undetermined legal responsibility (n = 10).

**Conclusions:**

We identified EPs’ and AHPs’ salient beliefs about using a wiki-based reminder. We will draw on these beliefs to construct a questionnaire to measure the importance of these determinants to EPs’ and AHPs’ intention to use a wiki-based reminder promoting evidence-based care for traumatic brain injuries.

## Introduction

As many as half of all patients with major traumatic injuries do not receive the recommended care [1-6]. Medical errors are common in critically ill trauma patients and mistakes occur frequently in emergency departments [6,7], where unconscious acts of omission and information overload [8,9] contribute to the problem. Indeed, emergency health care professionals must often make quick decisions, mostly based on intuitive reasoning [9]. Intuitive reasoning is fast, impulsive, effortless, and reflexive: while it serves the emergency health care professionals well, it is also prone to error. Reminders (eg, care protocols, order sets, and treatment algorithms) are knowledge tools that can improve intuitive decision making and help professionals implement best practices [9]. In particular, systematic reviews have indicated that computer reminders to health care professionals at the point of care can be effective at promoting best practices in a variety of clinical areas [10-19], including acute care, where they improve process-of-care outcomes [20]. Such reminders range from simple prescribing alerts to more sophisticated support for decision making. That said, different stakeholders have rejected many reminder systems on the grounds that they are slow, incompatible with work processes, unable to be adapted to local practices, difficult to access, or costly to implement [19]. A wiki, in contrast, stands as a low-cost means of permitting stakeholders from a single or many emergency departments to collaborate asynchronously on creating and updating reminders without duplicating their efforts or spending too much time.

A wiki is a webpage or a collection of webpages whose content can be modified by those who access it [21,22]. Wikis are being used to encourage and make it easier for researchers and clinicians working in medicine to share information and expertise [23-28]. Wikis can also help users adapt knowledge to local contexts within the knowledge-to-action cycle [19,29] and help patients and clinicians collaborate in developing patient support tools [30,31]. Studies have found that clinicians use reminders less than expected [32-35], but by giving clinicians an easy-to-use tool for creating, sharing, and updating reminders for their own and others’ use, wikis could reverse this trend. Wikis’ low cost makes them especially attractive. Clinical decision support systems that are not flexible enough to accommodate regular updating are very expensive [36,37]. For this reason, numerous authors have suggested exploring collaborative Web-based applications to share, create, and update clinical decision support content [19,36,38-40].

Although several wikis exist in health care, a wiki containing reminders to treat trauma patients does not exist yet. These wiki-based reminders for trauma would be created by a multidisciplinary group of clinicians interested in improving the quality of trauma care by implementing care protocols to help standardize trauma care. Using a wiki to share reminders could be a potentially powerful tool to allow a multidisciplinary group of health care professionals within the same hospital or situated in different hospitals to collaborate in the creation of high-quality, evidence-based reminders. The wiki would serve as a shared online repository available for health care professionals at the point of care.

Despite preliminary surveys showing health care professionals’ interest in wikis [28,41,42], very little is known about the determinants that influence professionals’ intention to use the information from wikis in clinical practice. To our knowledge, only one study [43] has explored the intention of physicians to use wikis to share medical information with other physicians. Although this study found that physicians were unsure about sharing medical information with other physicians, this behavior is different from using information from a wiki in clinical practice and has different determinants. Furthermore, trials exploring how eHealth applications—such as wikis—are used by patients have been plagued by high dropout rates, a phenomenon that the founding editor of this journal has termed the law of attrition [44]. No study has yet shown this phenomenon to apply to health professionals, but to minimize dropout in future trials exploring health professionals’ use of wiki-based reminders, we must design wikis that health professionals find helpful in caring for patients. To do this, we must understand what causes emergency physicians (EPs) and allied health professionals (AHPs) to use or to eschew the reminders. To this end, we plan to construct a questionnaire, based on the theory of planned behavior, that will ask health care professionals to quantify the influence of each determinant on their use of a wiki-based reminder in clinical practice. According to the theory of planned behavior, the constructs must be measured using a specific target, action, context, and time, as well as a specific population, to obtain results representative of that population [45]. The choice of trauma care for the setting of this study is linked to the investigators’ research interests and is only the first step in investigating the use of wiki-based reminders in other contexts and with different health care professionals. In particular, wiki-based reminder systems have the potential to support collaboration in clinical settings where multidisciplinary teams work together asynchronously (eg, group-based primary care). The results of this questionnaire will then help construct a theory-based intervention to increase the use of a wiki-based reminder by EPs and AHPs. This research project is the first step in that endeavor. Its goal is to solicit as complete as possible an array of beliefs concerning health professionals’ use of wiki-based reminders that promote best practices in trauma care, and to identify the most salient beliefs for inclusion in the questionnaire.

### Conceptual Underpinnings of the Proposed Study

**Figure 1:**
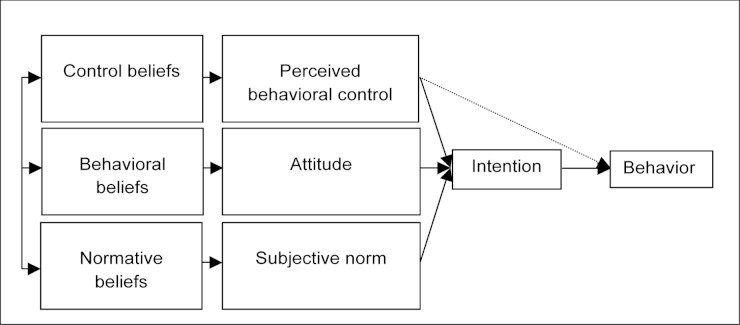
Theoretical framework of the theory of planned behavior.

The theory of planned behavior (Figure 1 [45,46]) is well known for its application to the study of health care professionals’ behaviors [47-56]. A recent systematic review has shown that Internet-based interventions based on the theory of planned behavior tend to have substantial effects on behavior [57]. This theory provides a theoretical account of the ways in which attitude, subjective norm, and perceived behavioral control combine to predict behavioral intention [55]. It postulates that when an individual has some control over a situation, intention is the immediate determinant of behavior [45]. Furthermore, if the individual’s perceived behavioral control reflects the individual’s actual behavioral control, this variable can be used to predict behavior directly.

Intention is influenced by three constructs: attitude, subjective norm, and perceived behavioral control. Attitude toward the behavior is a person’s evaluation of the consequences of adopting the behavior. Attitude is thus determined by the actor’s behavioral beliefs about the advantages and disadvantages of adopting the behavior. Subjective norm refers to perceived social pressure to engage or not to engage in a behavior. Subjective norm thus represents the actor’s normative beliefs—that is, his or her beliefs about how people who are in some way important to the actor would like the actor to behave. Finally, perceived behavioral control is the actor’s perception of how easy or difficult it is to perform the behavior. This perception is determined by the presence of perceived barriers and facilitators: control beliefs. According to Ajzen [45], an individual or a group’s salient beliefs are the beliefs that the individual or group reports most frequently with respect to the attributes of performing a particular behavior.

### Objectives

Our goal was to identify and compare the salient beliefs of EPs and AHPs about using a wiki-based reminder that promotes best practices in the management of patients with a severe traumatic brain injury in emergency departments in the province of Quebec, Canada.

## Methods

### Study Design

The detailed protocol of this research project has been published elsewhere [58]. Briefly, this paper presents the results of a qualitative survey that used semistructured interviews.

### Participants and Setting

The study took place in three officially designated trauma centers in the province of Quebec, Canada: a level I, a level II, and a level III trauma center (see Multimedia Appendix 1 for definitions). Our study involved EPs (excluding residents and medical students) and AHPs (nurses, respiratory therapists, and pharmacists) involved in planning and caring for trauma patients. We purposefully recruited participants to obtain a representative sample of professionals from each level of trauma center and to elicit the widest possible range of beliefs. Thus, we aimed to recruit 10 EPs and 10 AHPs in each of the level I and II centers and 5 EPs and 5 AHPs in the level III center. To recruit participants, we sent an email to the heads of each emergency department to help us target and obtain the email addresses of potential participants, and to help us target local opinion-leader health care professionals known to be reluctant to use computers and new technology, and members of local trauma committees responsible for monitoring the quality of care. We also intentionally recruited both junior and senior staff members and at least one member of each AHP profession from each trauma center. Following the department heads’ recommendations, we then contacted selected members of each unit within each trauma center by telephone, by email, or in person. Interviews were conducted on site. Our study was approved by the ethics review boards of all three hospitals.

### Data Collection Procedure

The data collection process began by each participant meeting a researcher (AB) individually to obtain informed consent. During this meeting, the researcher, who had conducted other surveys using the theory of planned behavior, explained the study process, outlined anonymity and confidentiality issues, and stated that the participant would not be remunerated for his or her time. All meetings were conducted in French and all took place in person except one, which took place by phone. After obtaining the participant’s consent, the researcher showed the participant a video that gave a brief, simple explanation of how different health care professionals could use wikis to collaborate in creating and updating wiki-based reminders for the care of traumatic brain injury patients. The video finished with a real-life enactment of the clinician performing the behavior of interest (using a wiki-based reminder) at a patient’s bedside. A different version of the video was produced for each professional group participating in the survey (physicians, nurses, respiratory therapists, and pharmacists; see Multimedia Appendix 2, Multimedia Appendix 3, Multimedia Appendix 4, and Multimedia Appendix 5 for original videos in French). The videos were adapted with permission from the *Wikis in Plain English *video created by Lee LeFever and Common Craft [59]. Two medical informatics experts (SC, MPD) ensured that the video appropriately described how the professional would incorporate the wiki-based reminder into his or her daily practice. After viewing the video, the participant read a clinical vignette that described in detail the behavior of interest presented in the video (using a wiki-based reminder in a typical case of severe traumatic brain injury) (see Multimedia Appendix 6). Vignettes are often used in qualitative research and may have various goals [60]. In this research, we used the vignette to make the participants think of a clinical encounter with a patient with a serious traumatic brain injury and to imagine how they would use the wiki-based reminder in a real-life situation. This helped prepare the participants for the semistructured questions about using a wiki-based reminder for their care of their patients. The clinical vignette was written with the help of three clinical experts, two of whom (JL, AL) were members of Quebec’s accreditation board for trauma centers (Trauma Care Continuum Assessment Team). The researcher then conducted a semistructured interview with the participant and elicited the participant’s feedback on three topics: (1) the advantages and disadvantages of the professional’s adoption of the behavior (behavioral beliefs), (2) influential people (referents) who would approve or disapprove of the participant’s adoption of the behavior (normative beliefs), and (3) barriers and facilitators to the professional’s adoption of the behavior (control beliefs). Each interview was digitally recorded, transferred to a computer, and transcribed. The interviewer also noted each participant’s answers on paper forms. All participants were assigned a number and remained anonymous. The voice recordings were heard only by people who were not acquainted with the respondents.

### Data Analysis

To identify participants’ beliefs, two researchers (AB, KA) experienced with the theory of planned behavior independently analyzed the contents of the interview transcripts and the notes taken during the interviews. Using deductive content analysis [61] as described in the theory of planned behavior, each researcher read the transcript of each interview to identify all of the beliefs expressed by each participant. Each belief was categorized in one of three Excel (Microsoft Corporation, Redmond, WA, USA) spreadsheets according to the type of belief (behavioral, normative, and control). Each belief was identified with a unique identifying number linking it to the questionnaire from which it was taken. The researchers also classified each belief as positive or negative (eg, ease of use vs time constraints) according to how the participant had perceived it in the interview. If some participants considered a belief to be positive while others considered it to be negative, the researcher classified the belief as positive if more respondents considered it positive than considered it negative, and classified it as negative in the opposite case. The two researchers then compared their findings to agree on the beliefs identified. This was done separately for EPs and AHPs, therefore creating a total of six lists.

The next step consisted of grouping the beliefs that expressed the same idea. This was done by the first researcher (AB) and validated by the second (KA). At this point, the researchers compared the beliefs with one another to remove duplicates. Dissent about grouping beliefs that expressed similar but slightly different ideas was resolved through discussion. When necessary, the principal investigator made the final decision.

Through discussion, the researchers then labeled the beliefs inductively without a predetermined taxonomy based on the ideas expressed by the participants. To better compare our results with the results of other studies of beliefs about the use of new information technology in health care, we appropriated some of the labels in the taxonomy developed by Gagnon et al [62]. We used these labels only when the beliefs identified in our survey were exactly the same as the beliefs described by Gagnon et al.

After having classified each belief in a category, marked it as positive or negative, and given it a label, we used the options in Excel to count the frequency of mentions of each belief. Within the three belief categories, we counted the total number of times each belief was reported by participants. Using Excel, we then ordered the beliefs from the most to the least frequently mentioned and assigned each belief a rank according to its position on this list. To identify the 75% most frequently mentioned beliefs for each category (behavioral, normative, and control), we divided the cumulative total number of mentions of each belief by the total number of mentions of all beliefs in that category and retained the top three-quarters as the salient beliefs for that category as per theory of planned behavior methodology [63]. If it was impossible to segregate precisely the top 75% of beliefs because certain beliefs occurred with the same frequency, we included all borderline beliefs (even if that meant retaining more than 75%) in order to represent participants’ beliefs comprehensively. We also retained less frequently reported beliefs that we felt could have an important influence on health care professionals’ use of wiki-based reminders. For the purpose of this paper, we translated each belief from French into English. As for the transcripts, we translated only those excerpts selected for publication.

To determine whether our participants’ baseline demographic characteristics were statistically different from those of the general population of health care professionals from which we had recruited our sample, we performed simple univariate statistical analyses. We used a 2-tailed Fisher exact test for dichotomous variables (gender) and a 2-tailed Student *t *test for continuous variables (age and years of experience). We used the free online statistical calculator GraphPad [64] to perform all statistical analyses.

## Results

### Participants’ Characteristics

**Figure 2:**
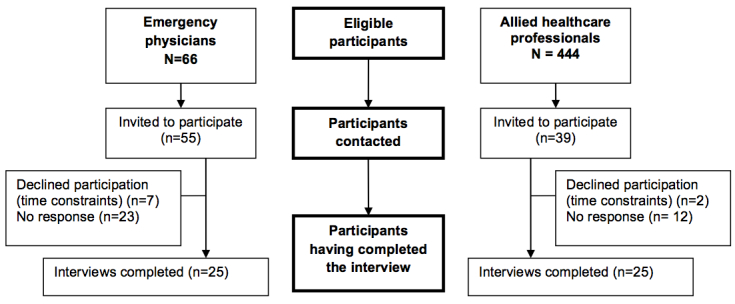
Flow of participants through study.

The three trauma centers comprised 66 eligible EPs and 444 eligible AHPs. To attain our target of 25 professionals per group, we invited 55 EPs and 39 AHPs to participate. Of these, 38% (25/66) of EPs and 6% (25/444) of AHPs agreed to take part. This translates to a response rate of 46% (25/55) for EPs and 64% (25/39) for AHPs. Of participating AHPs, 60% (15/25) were nurses, 28% (7/25) were respiratory therapists, and 12% (3/25) were pharmacists. These proportions are comparable with the distribution of eligible AHPs, of whom 57.7% (256/444) were nurses, 32.9% (146/444) were respiratory therapists, and 10% (42/444) were pharmacists. Our sample of EPs was composed of 10 EPs from a level I center, 10 from a level II center, and 5 from a level III center. Our sample of AHPs reproduced this distribution. Figure 2 illustrates the flow of participants in the study.

Compared with the 66 eligible EPs, the EPs who participated in this survey were similar in mean age (42 vs 43 years; *P *= .43), in mean years of experience (14 vs 16; *P *= .52), and in gender distribution (23/25, 92% vs 56/66, 85%). Compared with the 444 eligible AHPs, the AHPs who participated in the survey were also similar in mean age (38 vs 35 years; *P *= .19) and in gender distribution (18/25, 72% vs 372/444, 83.8%; *P *= .16). The only notable difference was that AHP participants had more clinical experience than average AHPs (14 vs 11 years; *P *= .04).

As shown in Table 1, which presents the participants’ characteristics, the EPs in our study constituted a diverse group of general practitioners and specialists in emergency medicine. The sample also covered a wide range of age groups and levels of clinical experience. The sample of AHPs was also composed of a wide range of professions, age groups, and levels of experience, with nurses constituting the largest number of professionals. Among the AHPs, 3 did not work in an emergency department, but either held responsibilities on the local trauma committee (1 nurse) or taught clinics to AHPs caring for patients with traumatic brain injury (1 nurse and 1 respiratory therapist). Our sample also included important decision makers who sat on the local trauma committee (4 EPs and 4 AHPs). All three trauma centers had Internet access in their emergency department and their resuscitation room.

Table 2 and Table 3 present the salient beliefs of EPs and AHPs, respectively. The tables contain verbatim examples for each belief. Tables 4 (Multimedia Appendix 7) and 5 (Multimedia Appendix 8) present the nonsalient beliefs.

**Figure 3:**
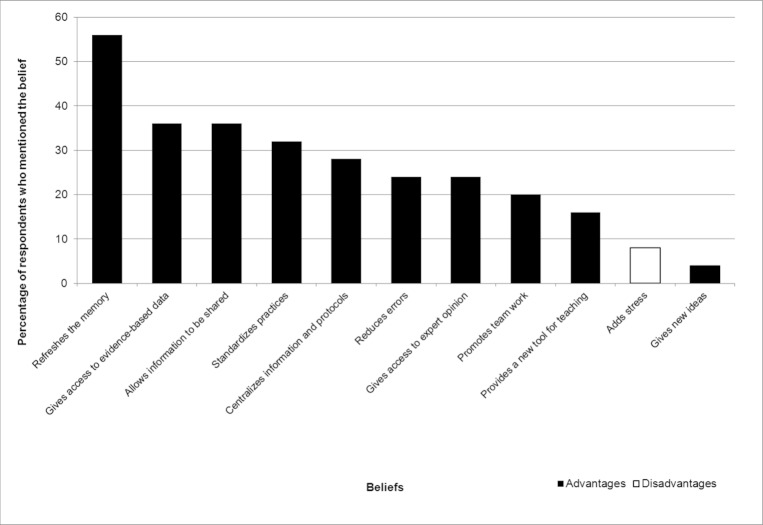
Proportion of emergency physicians who mentioned each behavioral belief (both salient and nonsalient).

**Figure 4:**
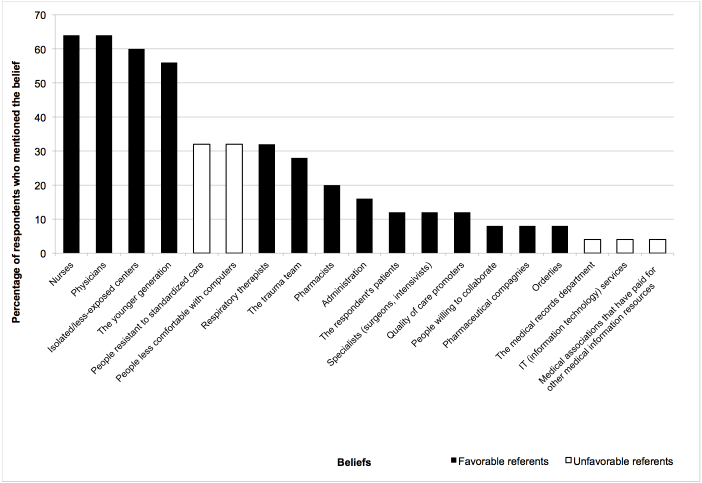
Proportion of emergency physicians who mentioned each normative belief (both salient and nonsalient).

**Figure 5:**
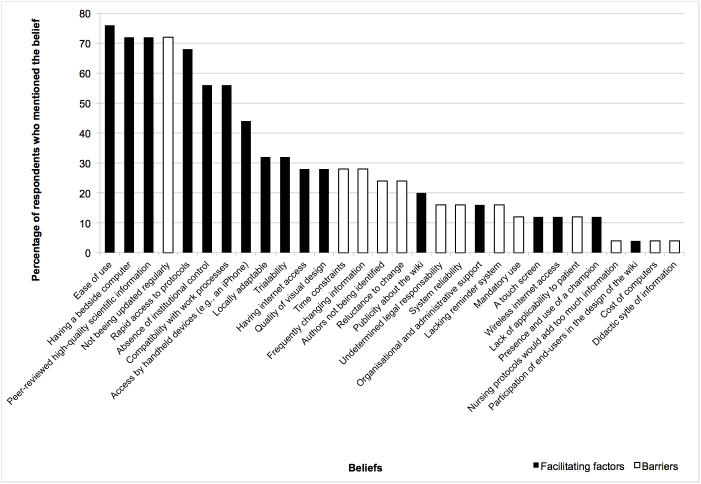
Proportion of emergency physicians who mentioned each control belief (both salient and nonsalient).

**Figure 6:**
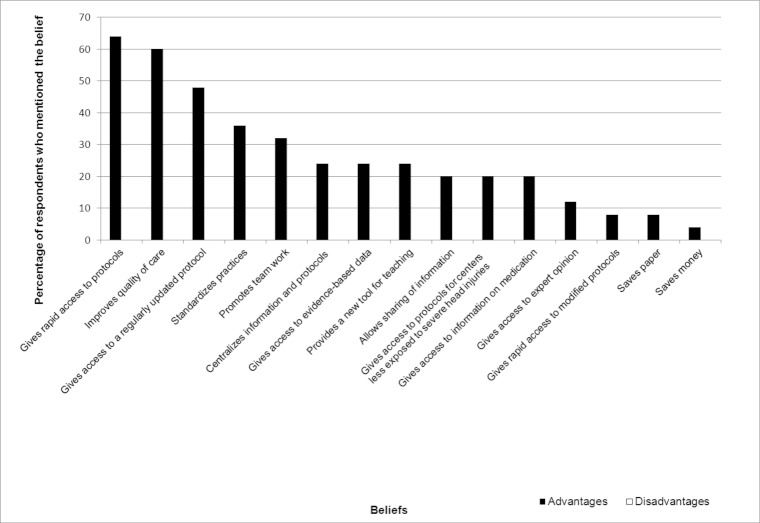
Proportion of allied health professionals who mentioned each behavioral belief (both salient and nonsalient).

**Figure 7:**
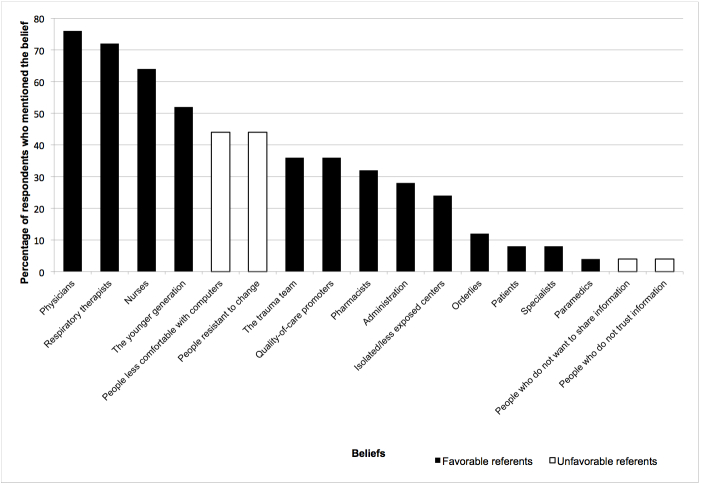
Proportion of allied health professionals who mentioned each normative belief (both salient and nonsalient).

**Figure 8:**
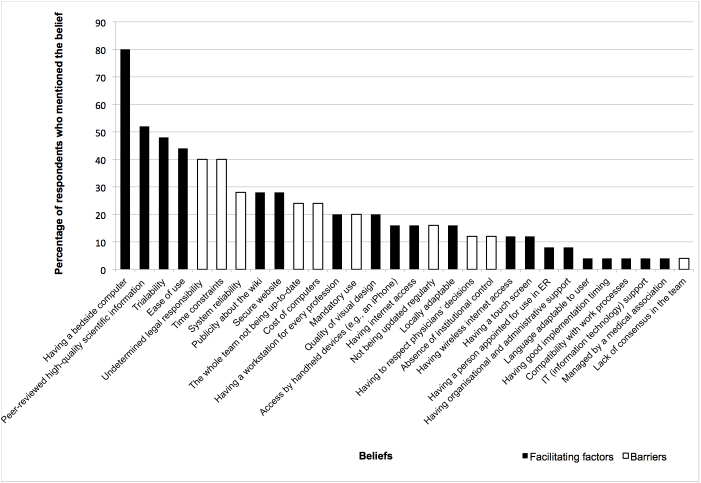
Proportion of allied health professionals who mentioned each control belief (both salient and nonsalient).

Figure 3, Figure 4, and Figure 5 display the percentages of EPs who had mentioned each belief (both salient and nonsalient) in each category, starting with the most frequently reported belief. Figure 6, Figure 7, and Figure 8 do likewise for AHPs.

**Table 1:** Characteristics of participating emergency physicians (EPs) and allied health professionals (AHPs).

Variable		EPs (n = 25)	AHPs (n = 25)
**Age (years)**			
	Mean (SD)	42 (9)	38 (10)
	Median (IQR)^a^	38 (35–49)	35 (29–44)
	Born between 1977 and 1997, n (%)	4 (16%)	10 (40%)
**Clinical experience (years)**			
	Mean (SD)	14 (10)	14 (9)
	Median (IQR)	11 (6–23)	11 (9–18)
**Gender, n (%)**			
	Male	23 (92%)	7 (28%)
**Emergency medicine certification, n (%)**			Not applicable
	College of Family Physicians (without emergency medicine certification)	9 (36%)	
	College of Family Physicians (with emergency medicine certification)	4 (16%)	
	Royal College of Physicians of Canada or Collège des médecins du Québec	8 (32%)	
**Profession, n (%)**		Not applicable	
	Nursing		15 (60%)
	Respiratory therapist		7 (28%)
	Pharmacist		3 (12%)
**Work environment, n (%)**			
	Emergency department	25 (100%)	22 (88%)
	Intensive care unit	2 (8%)	9 (36%)
	Member of a local or regional trauma committee	4 (16%)	4 (16%)
Previous use of a wiki^b^, n (%)		20 (80%)	15 (60%)
Previous use of Wikipedia^b^, n (%)		14 (56%)	8 (32%)
Previous editing of a wiki^b^, n (%)		1 (4%)	1 (4%)

^a ^Interquartile range.

^b ^We did not ask whether the respondent had used wikis for personal or for professional reasons.

**Table 2:** Emergency physicians’ salient beliefs about using a wiki-based reminder.

Rank^a^	Salient belief		n (%)^b^	Verbatim example
**Behavioral belief (n = 11)**				
	**Perceived advantage**			
1		Refreshes the memory	14 (20%)	“good revision”
2		Gives access to evidence-based data	9 (13%)	“see the best data”
3		Allows information to be shared	9 (13%)	“creates a collaborative space between hospitals”
4		Standardizes practices	8 (11%)	“consensus on the approach”
5		Centralizes information and protocols	7 (10%)	“prevents searching in different places”
6		Reduces errors	6 (8%)	“commit fewer mistakes”
7		Gives access to expert opinions	6 (8%)	“written by leaders in the field”
	**Perceived disadvantage**			
10		Adds stress^c^	2 (3%)	“stress is added by having to search information while your patient is there”
	Total		61/71 (86%)	
**Normative belief (n = 19)**				
	**Referents perceived as favorable**			
1		Nurses	16 (13%)	“nurses”
2		Physicians	16 (13%)	“physicians”
3		Isolated/less-exposed centers	15 (13%)	“centers less familiar with severe head injury”
4		The younger generation	14 (12%)	“the young”
7		Respiratory therapists	8 (7%)	“respiratory therapists”
8		The trauma team	7 (6%)	“the emergency team”
10		Administration^c^	4 (3%)	“the department heads”
11		The respondent’s patients^c^	3(3%)	“patients”
12		Specialists (surgeons, intensivists)^c^	3 (3%)	“specialties other than emergency”
	**Referents perceived as unfavorable**			
5		People resistant to standardized care	8 (7%)	“some people think that protocols are for robots”
6		People less comfortable with computers	8 (7%)	“people not comfortable using computers”
	Total		102/119 (86%)	
**Control belief (n = 31)**				
	**Perceived facilitating factor**			
1		Ease of use^d^	19 (8%)	“if it is user friendly, easy to navigate”
2		Having a bedside computer	18 (8%)	“must be easy to access directly in the resuscitation room”
3		Peer-reviewed high-quality scientific information	18 (8%)	“control over the quality of the information”
5		Rapid access to protocols	17 (7%)	“access should not take more than 3 clicks”
6		Absence of institutional control	14 (6%)	“having 18 passwords”
7		Compatibility with work processes^d^	14 (6%)	“integrated into daily work tools”
8		Access by handheld devices (eg, an iPhone)	11 (5%)	“available on handheld computers”
9		Locally adaptable	8 (4%)	“able to adapt it to the local flavor”
10		Trialability^d^	8 (4%)	“you have to use it often to become familiar”
11		Having Internet access	7 (3%)	“accessible from all locations by Internet”
12		Quality of visual design	7 (3%)	“the attractiveness of the site”
	**Perceived barrier**			
4		Not being updated regularly	18 (8%)	“if the protocol dates back and I know there are new data”
13		Time constraints^d^	7(3%)	“Not having the time, having to decide on the spot”
14		Frequently changing information	7 (3%)	“wiki always changing”
15		Authors not being identified^c^	6 (3%)	“be able to know who edited”
18		Undetermined legal responsibility^c^	4 (2%)	“who is ultimately legally responsible”
	Total		183/227 (81%)	

^a ^The rank number corresponds to the position held in the ranking of all beliefs. The most frequently mentioned belief is ranked first. The ranking numbers do not necessarily follow each other in this table, since we grouped them as advantages, disadvantages, favorable referents, unfavorable referents, barriers, and facilitators. These rank numbers correspond to their position in Figures 3 to 8.

^b ^n = the number of participants who reported the belief during their interview, and % = the number of times the belief was reported in all interviews divided by the number of times all beliefs in that category (behavioral, normative, and control beliefs) were reported in all interviews.

^c ^This belief was not mentioned in the top 75% most frequently reported but was retained nonetheless.

^d ^The label for this belief was taken from the Gagnon et al framework [62].

**Table 3:** Allied health professionals’ salient beliefs about using a wiki-based reminder.

Rank^a^	Salient beliefs		n (%)^b^	Verbatim example
**Behavioral belief (n = 15)**				
	**Perceived advantage**			
1		Gives rapid access to protocols	16 (16%)	“immediately available”
2		Improves quality of care	15 (15%)	“enhances the quality of care”
3		Gives access to a regularly updated protocol	12 (12%)	“always up-to-date”
4		Standardizes practices	9 (9%)	“everyone uses the same procedure”
5		Promotes team work	8 (8%)	“enables a multidisciplinary approach”
6		Centralizes information and protocols	6 (6%)	“able to consolidate the information”
7		Gives access to evidence-based data	6 (6%)	“based on evidence”
8		Provides a new tool for teaching	6 (6%)	“facilitates education”
	**Perceived disadvantage**			
		None perceived		
	Total		78/101 (77%)	
**Normative belief (n = 17)**				
	**Referents perceived as favorable**			
1		Physicians	19 (14%)	“physicians”
2		Respiratory therapists	18 (13%)	“respiratory therapists”
3		Nurses	16 (12%)	“nurses”
4		The younger generation	13 (9%)	“young people”
7		The trauma team	9 (7%)	“any professional working in the trauma bay with a [traumatic brain injury]”
8		Quality-of-care promoters	9 (7%)	“clinical coordinator”
10		Administration^c^	7(5%)	“general management”
	**Referents perceived as unfavorable**			
5		People less comfortable with computers	11 (8%)	“those with less computer skills”
6		People resistant to change	11 (8%)	“people less favorable to change”
	Total		113/137 (82%)	
**Control belief (n = 31)**				
	**Perceived facilitating factor**			
1		Having a bedside computer	20 (12%)	“have the computer close at hand”
2		Peer-reviewed high-quality scientific information	13 (8%)	“who ensures that the information is good”
3		Trialability^d^	12 (7%)	“must have training”
4		Ease of use^d^	11 (7%)	“simple, instinctive system”
8		Publicity about the wiki	7 (4%)	“should be publicized”
9		Secure website	7 (4%)	“secure system”
12		Having a workstation for every profession	5 (3%)	“each having a workstation”
14		Quality of visual design	5 (3%)	“simple presentation”
	**Perceived barrier**			
5		Undetermined legal responsibility	10 (6%)	“must know if the hospital endorses it”
6		Time constraints^d^	10 (6%)	“we must act, no time to go look”
7		System reliability	7 (4%)	“if the computer crashes”
10		The whole team not being up-to-date	6 (4%)	“if people are not up-to-date, could be difficult to apply it”
11		Cost of computers	6 (4%)	“the budget”
13		Mandatory use	5 (3%)	“it’s not because one center does it that way, that everybody should adopt that practice”
	Total		124/162 (77%)	

^a ^The rank number corresponds to the position held in the ranking of all beliefs. The most frequently mentioned belief is ranked first. The ranking numbers do not necessarily follow each other in this table, since we grouped them as advantages, disadvantages, favorable referents, unfavorable referents, barriers, and facilitators. These rank numbers correspond to their position in Figures 3 to 8.

^b ^n = the number of participants who reported the belief during their interview, and % = the number of times the belief was reported in all interviews divided by the number of times all beliefs in that category (behavioral, normative, and control beliefs) were reported in all interviews.

^c ^This belief was not mentioned in the top 75% most frequently reported but was retained nonetheless.

^d ^The label for this belief was taken from the Gagnon et al framework [62].

### Behavioral Beliefs: Advantages and Disadvantages

The three behavioral beliefs about using a wiki-based reminder that EPs mentioned most frequently were, in order of frequency, that if refreshes the memory, gives access to evidence-based data, and allows information to be shared. No disadvantages figured in the top 75% of beliefs, and only one disadvantage was reported at all, with 2 EPs opining that a wiki-based reminder system would add the stress of having to look for information while the patient was there in front of them (Table 2). We retained this belief as salient because it was the only disadvantage reported. The three behavioral beliefs about using a wiki-based reminder that AHPs mentioned most frequently were that it gives the user rapid access to protocols, improves the quality of care, and gives the user access to a regularly updated protocol. AHPs reported no salient disadvantages (Table 3).

### Normative Beliefs: Positive and Negative Referents

The three referents most cited by EPs as likely to approve or disapprove of the behavior were nursing personnel, physicians, and isolated or less-exposed trauma centers. All were seen as favorable to the respondent’s adopting the behavior (Table 2). The three referents most often mentioned by AHPs were physicians, respiratory therapists, and nurses, all of whom were also considered to approve of the behavior. For EPs, we retained as salient two beliefs pertaining to referents who would disapprove of the behavior: people resistant to standardized care and people less comfortable with computers. AHPs also stated that people less comfortable with computers would not approve. In addition, AHPs feared that people resistant to change would not approve (Table 3). We also retained three beliefs not mentioned in the top 75% for EPs—namely, the respondent’s patients, the administration, and specialists—because we consider that these referents play an important role in implementing reminders promoting best practices in trauma care [65-67] and in implementing information and communication technology (ICT) [62,68-70]. We also retained administration for AHPs for the same reasons.

### Control Beliefs: Facilitating Factors and Barriers

EPs indicated that the three top facilitators were ease of use, having a computer at the bedside, and accessing information that was peer reviewed and of high scientific quality (Table 2). The most frequently reported barrier was the wiki-based reminder not being regularly updated; the next most frequently reported barriers were time constraints and frequently changing information. We selected as salient beliefs two barriers that were not reported in the top 75%: authors not being identified and undetermined legal responsibility. We also selected these beliefs because they have been frequently reported by other authors [32,36,39,71].

Among AHPs, the three most frequently mentioned facilitators were having a computer at the bedside, accessing information that was peer reviewed and of high scientific quality, and trialability (how easy it is to experiment with the tool) (Table 3). The three most frequently mentioned barriers reported by AHPs were the undetermined legal responsibility of using a wiki, time constraints, and an unreliable information system.

## Discussion

This study identified EPs’ and AHPs’ beliefs about using a wiki-based reminder to promote best practices in caring for patients with a severe traumatic brain injury. Based on the theory of planned behavior, we categorized these beliefs as behavioral, normative, and control beliefs. After analyzing the beliefs and ranking them in order of frequency of mention, we labeled the 75% most-reported beliefs as salient. We also labeled salient certain beliefs that were not among the 75% most reported. This post hoc decision was based on our knowledge of the literature (eg, “administration” as a normative belief), our experience in implementing care protocols for trauma (eg, “specialists” as a normative belief), or our fear of excluding important negative beliefs (eg, “adds stress” as a behavioral belief).

EPs and AHPs saw many of the same advantages to using a wiki-based reminder: namely, that a reminder gives access to evidence-based data, that it standardizes practices, and that it centralizes protocols. EPs and AHPs also shared similar normative beliefs about parties favorable to the use of a wiki-based reminder (nurses, physicians, respiratory therapists, the trauma team, and the younger generation), and both groups mentioned people less comfortable with computers as a negative referent. Many facilitating factors were common to EPs and AHPs: having a computer at the bedside, accessing information that was peer reviewed and of high scientific quality, ease of use, trialability, and an attractive visual design. The groups also had two obstacles in common: time constraints and undetermined legal responsibility.

Our two groups of respondents also differed in the perceived advantages to using a wiki-based reminder. While both saw the centralization of information and protocols as an advantage, only EPs saw the sharing of information as an advantage and only AHPs saw the promotion of teamwork as an advantage. Similarly, both groups saw easy access to a wiki-based reminder (eg, having a bedside computer) as a characteristic that would make using the reminder simple to use, but only EPs saw rapid access to protocols (“fewer than three clicks”) as a simplifying feature, and only AHPs saw having a workstation for every professional as such a feature. The apparent contradiction between the AHPs’ concern about having a secure website and the EPs’ desire to avoid passwords is worth exploring. Finally, AHPs felt it important to publicize and otherwise promote the wiki-based reminder to make it more visible (or in Rogers’s terms, which we explain below, “observable”). AHPs saw this as important to the innovation’s uptake, recognizing that the more people observe others using a wiki, the more likely they are to use it too.

We noted other differences. Significantly, AHPs named mandatory use as a dominant barrier. At the same time, AHPs often referred to EPs, quality-of-care promoters, and hospital administrators as important decision makers in the care of patients with a severe traumatic brain injury. Thus, if EPs, quality-of-care promoters, and hospital administrators make it mandatory to use a wiki-based reminder, the importance of this barrier might decrease. Research suggests that individuals are more likely to comply with referents’ expectations when the referents in question can reward or punish nonbehavior, as is often the case in a mandatory setting [72,73]. Future work will have to measure and compare the relative importance of these beliefs. These measurements will be used to determine whether implementation strategies should be adapted to different groups of professionals.

Many of the findings in our study confirm the findings of authors who have studied the adoption of other ICTs and of innovations in general. For instance, our participants reported compatibility with work process and trialability as important beliefs. In the diffusion of innovation theory, Rogers identifies these two characteristics in addition to three others—the innovation’s relative advantage, its complexity or simplicity, and its observability (the degree to which it is visible to users and potential users)—as influencing an individual’s decision to adopt or reject an innovation [74]. Authors besides Rogers have also associated an ICT’s lack of compatibility with work process and its poor trialability with the innovation’s unsuccessful implementation [32,39,75,76]. Because wiki-based reminders are designed to facilitate changes and edits by all users, they can be modified to fit different work processes [32,36,39]. Likewise, wikis’ free and open access could facilitate their trialability [41,42,71]. Hence, these seem like important advantages whose influence will need to be measured in future studies. Our participants reported the other three diffusion of innovation characteristics indirectly.

The findings of this survey are also consistent with the factors proposed by Davis [77] as determinants of the adoption of technology in his Technology Acceptance Model. In Davis’s model, the system’s perceived ease of use and its perceived usefulness were among the most frequently mentioned beliefs. Similarly, ease of use was the EPs’ top control belief and the AHPs’ fourth most frequently mentioned belief. As for usefulness, salient beliefs not yet mentioned in this discussion included that a reminder reduces errors (EPs), that it gives users rapid access to protocols (AHPs), that it gives users access to regularly updated protocols (AHPs), and that it is a new teaching tool (AHPs). Prospective measurement of the influence of these beliefs will be important, as high perceptions of system usefulness and ease of use have been associated with cases of ICT adoption [62].

Our study identified additional beliefs (aside from those similar to the beliefs identified by Rogers and by Davis) that were identical to the barriers and facilitators found in a recent systematic review of factors influencing health care professionals’ adoption of ICTs [62]. The beliefs in question were the presence and use of a champion, the participation of end users, and time constraints. Time constraints in particular have been identified as an important control belief in studies on ICT adoption [32,41,62,71,78] and in other contexts as well [79]. This is why we considered it salient, even though it ranked only 13th for EPs and sixth for AHPs. Other salient beliefs concerned the speed with which the user could access the reminder (the fifth-ranked control belief for EPs) and the rapidity with which the reminder gave the user access to protocols (the top-ranked behavioral belief for AHPs). Other control beliefs identified in our study were also similar to those identified in the systematic review mentioned above [62]: the quality of the visual design (salient for both groups), the absence of institutional control (salient for EPs, nonsalient for AHPs), the reminder’s reliance on peer-reviewed information of high scientific quality (salient for both groups), the mandatory use of the reminder (salient for AHPs, nonsalient for EPs), the lack of a reminder to use the wiki (nonsalient for EPs), having a computer at the bedside (salient for both groups), adding stress (salient for EPs), and use by people less comfortable with computers (salient for both groups).

We also found similarities to studies on the adoption of a computerized decision support system. In these studies, clinicians most wanted such a system to remind them of what they already intended to do [19,34,80,81]. Similarly, the EPs’ most frequently reported behavioral belief was that using a wiki-based reminder would refresh their memory. Centralized information (the EPs’ fifth-ranked salient behavioral belief) and access from different areas of the hospital have also been described in the literature as important factors in using a computerized decision support system [38,39]. Respondents also perceived these systems as improving patient care [80]: this is similar to the EPs’ belief that a wiki-based reminder would help reduce errors (sixth salient behavioral belief).

Our study of wikis also confirmed several barriers described in studies of health care professionals’ beliefs about using social media [32,36,39,41-43,78]: concern about the quality of information (EPs and AHPs), undetermined legal responsibilities [36,39,71] (EPs and AHPs), and lack of author identification [32,36] (EPs only). Measuring professionals’ perceptions of the importance of these barriers in our questionnaire will be essential to determine how these barriers might influence the use of a wiki-based reminder.

This overlap between study findings, notwithstanding our rigorous use of a theoretical framework, allowed us to identify new beliefs specific to our target population and related to the adoption of wiki-based reminders, beliefs that studies of the adoption of social media in health care had not identified. The importance of these new beliefs will also be important to measure in a future questionnaire. In terms of behavioral beliefs, both EPs and AHPs reported that using a wiki-based reminder could help standardize practices, promote teamwork (salient only for AHPs), and give users access to regularly updated protocols. EPs did not perceive this last factor as an advantage, but stated that having a system that was updated regularly would be a facilitating factor. Surprisingly, EPs also stated that frequently changing information would be a barrier. The importance of these apparently conflicting beliefs will be important to measure because reminders contained within a wiki could indeed change quite frequently if the literature changes frequently or if an edit war should occur. An edit war arises when a user repeatedly re-edits, undoes, or reverses a prior user’s edits in an attempt to keep visible his or her preferred version of a page [82].

We also identified many influential groups of people who would be favorable to health care professionals’ use of a wiki-based reminder, with EPs and AHPs naming each other as their main influence. So far, interpretations of the role of social influence on the adoption of ICTs have varied. Some authors have argued for the inclusion of normative beliefs (sources of social influence) in models of adoption and use [83,84], while others have excluded them [77]. Furthermore, some work has found that social influence is significant only under certain circumstances: in settings where ICT use is mandatory [71,85], among women in the early stages of their experience [86,87], and among older workers [88]. However this may be, we believe our study to be the first to have rigorously identified health care professionals’ salient normative beliefs concerning the use of any form of social media in health care. This is significant, because understanding the influence of normative beliefs on health professionals’ intentions to use social media such as wikis will be of the essence: social media are hypothesized to operate based on social networking, participation, collaboration, apomediation, and openness between peers [89]—all elements related to social influence.

Although age had been noted as a moderating factor in predicting the adoption of ICTs [87], past studies have not described the “younger generation” as an influential referent group, as AHPs and EPs did in our study. This finding is significant, since members of generation Y (people born between 1977 and 1997) will soon constitute a major part of the health care workforce and have been described as being comfortable with technology [90].

Some EPs working in the level I trauma center expressed another important normative belief. They suggested that clinicians in level II and III centers would be more likely to use a wiki-based reminder for the care of patients with a severe traumatic brain injury than would experienced clinicians working in level I centers, who would not need to refer to a reminder. This suggestion reflects the fact that many clinicians working in level I trauma centers view themselves as leaders and champions who help less-experienced clinicians better manage traumatic brain injuries. The influence of this belief must be measured quantitatively. If the experts do not intend to use wiki-based reminders themselves, future exploration must verify whether they intend to contribute their expertise to a wiki in order to help staff at level II and III centers improve their practices. It seems not unlikely that when recognized experts and strong leaders in a field add material to a wiki of evidence-based reminders promoting best practices, other health care professionals are motivated to take up the material in question. Further exploration of the intention to contribute medical information to a wiki will be particularly important because it seems that the level of sharing of medical information through social media is lower than expected for health care professionals [28,43,78].

In spite of its rigorous methods, our study has limitations. First, we did not perform member checking, even though member checking would have made our results more credible. However, two independent research professionals experienced with using the theory of planned behavior analyzed the contents of the interview transcripts rigorously to interpret our respondents’ beliefs as trustworthily as possible and resolved disagreements by referring scrupulously to the transcripts. Furthermore, to make it possible for readers to interpret the results for themselves, we have presented a sample transcript for each salient belief.

A second limitation is that our survey was conducted with a small group of EPs and AHPs who were recruited from a single region of the province of Quebec. Even though Godin and Kok [50] suggest that a sample of 25 participants is sufficient to elicit salient beliefs, we cannot assume that our results are transferable to all clinicians, especially in the case of AHPs, where no one professional group (nurses, respiratory therapists, or pharmacists) was sufficiently represented. Nonetheless, even if we surveyed only 6% (25/444) of all AHPs, the proportion of each professional group within our sample was very similar to the proportion of those groups within the total population of eligible AHPs in the three trauma centers from which we drew the sample. Finally, we made every attempt to elicit various beliefs by purposefully sampling participants from three levels of trauma centers, with varying levels of experience, and by including clinicians known to be reluctant to use computers and ICTs. We believe that this sampling method resulted in our recruiting AHPs who were more experienced than the norm. Even though it is difficult to predict the influence of this selection bias, we deliberately chose to have more experienced clinicians take part in this survey, as they are known to have more negative views about adopting new technologies [88]. In predicting the adoption of wiki reminders, measuring negative views is just as important as measuring positive ones.

Third, the video we produced to describe the behavior of study portrayed wikis positively and could have influenced interviewees’ beliefs positively as well. Using a mix of animation and actors, the 6-minute video presented wikis as a new and effective way of communicating and collaborating. We produced this video because we needed to demonstrate in a short time how a wiki-based reminder could be used to improve the care of trauma patients and because the use of a wiki-based reminder to promote best practices in trauma remains theoretical for most health care professionals. It also involves many smaller lead-in behaviors (eg, connecting to the Internet, reading the reminder, and applying its suggestions) that are hard to explain during an interview. This said, after realizing that most of the beliefs expressed by participants after viewing the video were positive, we compensated for this possible bias by including negative beliefs that did not meet our 75% most frequently mentioned criteria in our list of salient beliefs.

Finally, our behavior of study was only the use of a wiki-based reminder, not the creation and editing of the reminder. The last two behaviors are important to study in order to understand how a wiki can fulfill its potential to turn a traditionally passive consumer of knowledge into an active producer of knowledge (or *prosumer*, in Eysenbach’s terms) [89].

A major strength of this study was its rigorous application of the methods proposed by the authors of the theory of planned behavior to assess our target population’s perceived behavioral, normative, and control beliefs related to using a wiki-based reminder. These beliefs will be used to construct a theory-based intervention to increase the use of a wiki-based reminder by EPs and AHPs. Using this theory facilitated our comparison with similar studies and contributed to our elaboration of a theoretical basis for understanding the decision making leading to this behavior. It will also allow researchers to carry out a systematic review in this area. Furthermore, the steps taken to analyze the content of beliefs are rarely described in detail. Our detailed and rigorous description of the content analysis makes it possible for other researchers to reproduce this approach to exploring health care professionals’ salient beliefs about the use of other social media in health care.

### Conclusion

This theory-based study has systematically identified the beliefs underlying EPs’ and AHPs’ intention to use information from a wiki when caring for patients with a traumatic brain injury. It is the first step in our attempt to understand EPs’ and AHPs’ intentions to use such a reminder, and will help us construct a validated questionnaire that will survey a broader population of EPs and AHPs about their intention to use wiki-based reminders promoting evidence-based traumatic brain injury care. By identifying the most important determinants of EPs’ and AHPs’ intention to use a wiki-based reminder, we will better understand how wikis could act as knowledge translation interventions to increase evidence-based practices in this area.

## Multimedia Appendix 1

Description of services offered at each level of trauma center.

## Multimedia Appendix 2

Video for emergency physicians.

## Multimedia Appendix 3

Video for nursing personnel.

## Multimedia Appendix 4

Video for respiratory therapists.

## Multimedia Appendix 5

Video for pharmacists.

## Multimedia Appendix 6

Clinical vignette and semistructured questionnaire.

## Multimedia Appendix 7

Table 4. Emergency physicians’ nonsalient beliefs about using a wiki-based reminder.

## Multimedia Appendix 8

Table 5. Allied health professionals’ nonsalient beliefs about using a wiki-based reminder.

## Abbreviations

AHP: Allied health care professional
EP: Emergency physician
ICT: information and communication technology
