# Supplementary material for: Health Care Professionals’ Beliefs About Using Wiki-Based Reminders to Promote Best Practices in Trauma Care
Source: J Med Internet Res. 2012 Apr 19;14(2):e49. doi: 10.2196/jmir.1983 (PMC3376518; doi:10.2196/jmir.1983)
Supplement: Supplementary file 1 [file jmir_v14i2e49_app1.pdf]

Appendix 1

Description of services offered at each level of trauma centre

The **level I** trauma center offers the full scope of definitive trauma care, including neurosurgery, general surgery, and orthopedic surgery. It runs an intensive care unit staffed by full-time certified intensivists, and an emergency department staffed by EPs certified by the Royal College of Physicians and Surgeons of Canada, the College of Family Physicians of Canada, or the Collège des Médecins du Québec.

The **level II** trauma center offers full-time, year-round coverage of orthopaedic surgery and general surgery, and runs an intensive-care unit staffed by full-time certified intensivists and an emergency department staffed by certified EPs.

The **level III** trauma center offers full-time, year-round coverage of general surgery and partial coverage of orthopaedic surgery; it runs an emergency department staffed by general practitioners. It has an intensive care unit, but it is not run by full-time certified intensivists.
