# Supplementary material for: Health Care Professionals’ Beliefs About Using Wiki-Based Reminders to Promote Best Practices in Trauma Care
Source: J Med Internet Res. 2012 Apr 19;14(2):e49. doi: 10.2196/jmir.1983 (PMC3376518; doi:10.2196/jmir.1983)
Supplement: Supplementary file 6 [file jmir_v14i2e49_app6.pdf]

Clinical vignette (presented after viewing the video)

YOU ARE A HEALTH PROFESSIONAL WORKING IN A TRAUMA CENTER OF THE PROVINCE OF QUEBEC. YOU ARE IN THE EMERGENCY DEPARTMENT WITH A SEVERE TRAUMATIC BRAIN INJURY (TBI) VICTIM. YOU WANT TO USE A REMINDER TO HELP YOU PROVIDE THE BEST CARE POSSIBLE FOR THIS SEVERE TBI VICTIM. WITHIN THE WIKI, "WIKI-EMERGENCY", A REMINDER ON THE INITIAL MANAGEMENT OF SEVERE TBI IN THE EMERGENCY DEPARTMENT CREATED BY SOME COLLEAGUES OF A LEVEL I TRAUMA CENTER IS AVAILABLE. THE REFERENCES SUPPORTING THE REMINDER ARE AVAILABLE AND YOU EVEN NOTICE THAT THE REMINDER WAS REVISED IN OCTOBER 2009 USING THE PRACTICAL GUIDE FROM THE 2007 BRAIN TRAUMA FOUNDATION AND USING THE LATEST EVIDENCE-BASED DATA FOUND IN THE LITERATURE.

PLEASE TAKE A FEW MINUTES TO THINK ABOUT THE FOLLOWING QUESTIONS:

1. What do you see are the advantages of using a wiki-based reminder that promotes best practices in the management of victims of a severe traumatic brain injury (TBI) in emergency departments in the province of Quebec?
2. What do you see are the disadvantages of using a wiki-based reminder that promotes best practices in the management of victims of a severe traumatic brain injury (TBI) in emergency departments in the province of Quebec?
3. Who are the individuals or groups of people who could be favorable to you using a wiki-based reminder that promotes best practices in the management of victims of a severe traumatic brain injury (TBI) in emergency departments in the province of Quebec?
4. Who are the individuals or groups of people who could be unfavorable to you using a wiki-based reminder that promotes best practices in the management of victims of a severe traumatic brain injury (TBI) in emergency departments in the province of Quebec?
5. What would help you use a wiki-based reminder that promotes best practices in the management of victims of a severe traumatic brain injury (TBI) in emergency departments in the province of Quebec?
6. What would prevent you from using a wiki-based reminder that promotes best practices in the management of victims of a severe traumatic brain injury (TBI) in emergency departments in the province of Quebec?
7. Are there any other elements that you associate with using a

wiki-based reminder that promotes best practices in the management of victims of a severe traumatic brain injury (TBI) in emergency departments in the province of Quebec?

Age: ______________________ Gender: ____________________

What is your work environment?

Emergency room   Operation   Critical care   Radiology department   Other: _________________

Hospital: ____________________________

Years of experience: _____________________

Profession: ____________________ Diploma: ________________________

Emergency medicine training: none   CFPC*   RCPSC*

*CFPC: The College of Family Physicians of Canada, *RCPSC: Royal College of Physicians and Surgeons of Canada

Number of severe traumatic brain injury victims per year: _______________

Previous consultation of another wiki (e.g., Wikipedia):

Yes   No If so, which one: ___________________________

Previous edition of a wiki page (e.g., Wikipedia):

Yes   No If so, which one: ___________________________

Member of local trauma care council: Yes

No

Presence of a computer with internet access in your emergency room:

Yes No

If so, in the resuscitation room? Yes No

Thank you for your cooperation!
