# Supplementary material for: Health Care Professionals’ Beliefs About Using Wiki-Based Reminders to Promote Best Practices in Trauma Care
Source: J Med Internet Res. 2012 Apr 19;14(2):e49. doi: 10.2196/jmir.1983 (PMC3376518; doi:10.2196/jmir.1983)
Supplement: Supplementary file 7 [file jmir_v14i2e49_app7.pdf]

Table 4. EPs' non-salient beliefs about using a wiki-based reminder

Rank (n=11)	Non-salient behavioral beliefs	n (%)[a]	Verbatim examples
	Perceived advantages:		
8	Promotes team work	5 (7)	"makes things easier for the team"
9	Provides a new tool for teaching	4 (6)	"training with residents, students"
11	Gives new ideas	1 (1)	"for new ideas"
	***Perceived disadvantages*:**		
	None		
**Rank (n=19)**	**Non-salient normative beliefs**	**n (%)[a]**	**Verbatim examples**
	Referents perceived as favorable:		
9	Pharmacists	5 (4)	"pharmacists"
10	Administration	4 (3)	"administrative authorities and heads of department"
13	Quality of care promoters	3 (3)	"people responsible for quality"
14	People willing to collaborate	2 (2)	"people with collaborative minds"
15	Pharmaceutical companies	2 (2)	"pharmaceutical companies"
16	Orderlies	2 (2)	"orderlies"
	Referents perceived as unfavorable:		
17	The medical records department	1 (1)	"archives"
18	IT (information technology) services	1 (1)	"IT services"
19	Medical associations that have paid for other medical information resources	1 (1)	"comes into direct competition with point of care products paid by medical association"
**Rank (n=31)**	**Non-salient control beliefs**	**n (%)[a]**	**Verbatim examples**
	Perceived facilitating factors:		
17	Publicity about the wiki[b]	5 (2)	"promote it"
20	Organisational and administrative support	4 (2)	"need the government to be a stakeholder"
23	A touch screen	3 (1)	"touch screen"
24	Wireless internet access	3 (1)	"available by WIFI internet access"
26	Presence and use of a champion[b]	3 (1)	"need a wiki representative"
29	Participation of end-users in the design of the wiki[b]	1 (1)	"see what people think and involve them in the process"
	Perceived obstacles:		
16	Reluctance to change	6 (3)	"breaking the psychological barrier can be difficult"
19	System reliability[b]	4 (2)	"technical problems"
21	Lacking reminder system	4 (2)	"reminders that it exists"
22	Mandatory use	3 (1)	"risk of being put in a straitjacket"
25	Lack of applicability to patient	3 (1)	"my case is different from the case before, it will not apply"

28	Nursing protocols would add too much information	1 (1)	“nurses have their protocols, could become overloaded”
30	Cost of computers	1 (1)	“funds for computers”
31	Didactic style of information	1 (1)	“information for revision, not for learning”

[a] n=the number of participants who reported the belief during their interview. %=the number of times the belief was reported in all interviews divided by the number of times all beliefs of that category (behavioral, normative and control beliefs) were reported in all interviews.
[b] The name of this belief was taken from the Gagnon et al framework [62].
