# Supplementary material for: Health Care Professionals’ Beliefs About Using Wiki-Based Reminders to Promote Best Practices in Trauma Care
Source: J Med Internet Res. 2012 Apr 19;14(2):e49. doi: 10.2196/jmir.1983 (PMC3376518; doi:10.2196/jmir.1983)
Supplement: Supplementary file 8 [file jmir_v14i2e49_app8.pdf]

Table 5. AHPs' non-salient beliefs about using a wiki-based reminder

Rank (n=15)	Non-salient behavioral beliefs	n (%)[a]	Verbatim examples
	Perceived advantages:		
9	Allows sharing of information	5 (5)	"allows exchanges, validation of certain practices"
10	Gives access to protocols for centers less exposed to severe head injuries	5 (5)	"makes the life easier for those who don't often receive some"
11	Gives access to information on medication	5 (5)	"always good to know which medication is preferable"
12	Gives access to expert opinion	3 (3)	"protocol validated by experts"
13	Gives rapid access to modified protocols	2 (2)	"no need to wait after the person in charge"
14	Saves paper	2 (2)	"saves a lot of paper"
15	Saves money	1 (1)	"saves a lot of money"
	Perceived disadvantages		
	None perceived		
**Rank (n=17)**	**Non-salient normative beliefs**	**n (%)[a]**	**Verbatim examples**
	Referents perceived as favorable:		
9	Pharmacists	8 (6)	"pharmacists"
10	Administration	7 (5)	"general management"
11	Isolated/less exposed centers	6 (4)	"small regional centers"
12	Orderlies	3 (2)	"orderlies"
13	Patients	2 (1)	"patients"
14	Specialists	2 (1)	"specialists"
15	Paramedics	1 (1)	"paramedics"
	Referents perceived as unfavorable:		
16	People who do not want to share information	1 (1)	"people who do not want to share information, to keep it for themselves"
17	People who do not trust information	1 (1)	"people who do not trust information"
**Rank (n=30)**	**Non-salient control beliefs**	**n (%)[a]**	**Verbatim examples**
	Perceived facilitating factors:		
15	Access by handheld devices (e.g., an iPhone)	4 (2)	"having a handheld device"
16	Having internet access	4 (2)	"having internet in the resuscitation room"
18	Locally adaptable	4 (2)	"the reality of one center is not necessarily the same for another"
21	Having wireless internet access	3 (2)	"wireless network"
22	Having a touch screen	3 (2)	"touch screen"
23	Having a person appointed for use in ER	2 (1)	"somebody assigned to read out loud"
24	Having organisational and administrative support	2 (1)	"comes from an organisational decision"
25	Language adaptable to user	1 (1)	"adapted to the language of the user"
26	Having good implementation timing	1 (1)	"good timing to implement"

27	Compatibility with work processes[b]	1 (1)	“integrated to daily work tools”
28	IT (information technology) support	1 (1)	“good IT support”
29	Managed by a medical association	1 (1)	“if it is managed by a medical association, I have more trust”
	Perceived obstacles:		
17	Not being updated regularly	4(2)	“information is not up-to-date”
19	Having to respect physicians’ decisions	3 (2)	“I don’t make the decisions”
20	Absence of institutional control	3 (2)	“if we have to log on”
30	Lack of consensus in the team	1 (1)	“if there is no consensus in the team”

[a] n=the number of participants who reported the belief during their interview. %=the number of times the belief was reported in all interviews divided by the number of times all beliefs of that category (behavioral, normative and control beliefs) were reported in all interviews.
[b] The name of this belief was borrowed from the Gagnon et al framework [62].
